# The relationship between oxidative balance score and erectile dysfunction in the U.S. male adult population

**DOI:** 10.1038/s41598-024-61287-w

**Published:** 2024-05-10

**Authors:** Mutong Chen, Zhongfu Zhang, Rui Zhou, Baizhi Li, Jiahao Jiang, Bentao Shi

**Affiliations:** 1 Department of Urology, The First Affiliated Hospital of Shenzhen University, Shenzhen Second People’s Hospital, Shenzhen, China; 2 Clinical Psychology/Psychosomatic Medicine Department, Shenzhen People’s Hospital, Shenzhen, China; 3 Shantou University Medical College, Shantou, China

**Keywords:** Oxidative balance score, Erectile dysfunction, Oxidative stress, Cross-sectional study, Erectile dysfunction, Risk factors, Lifestyle modification, Epidemiology

## Abstract

Oxidative stress strongly influences the pathophysiology of erectile dysfunction (ED). In this study, we used the oxidative balance score (OBS), a composite index, to measure the effects of oxidative stress triggered by diet and lifestyle factors. Here, we conducted a cross-sectional study to determine the statistical relationship between OBS and ED among adult males in the U.S. The data from 3318 participants in the National Health and Nutrition Examination Survey (NHANES) 2001–2004 were analyzed. Weighted logistic regression was used to correct for confounding factors and acquire nationwide representative estimates. Generalized additive modeling was used to explore the nonlinear relationship. We also supplemented subgroup and sensitivity analysis to examine the robustness of the main results. Multivariate logistic regression indicated a consistent negative linear association between OBS and ED across all participants [OR (95% CI) = 0.96 (0.94, 0.98)]. After categorizing OBS into tertiles, participants in the highest tertile had 43% lower odds of having ED than those in the lowest tertile [OR (95% CI) = 0.57 (0.37, 0.87)]. The generalized additive model also visualized the linear trend of this association. Furthermore, this linear relationship remained relatively consistent, regardless of whether subgroup or sensitivity analyses were performed. Our findings suggest that adopting a lifestyle and diet pattern that promotes favorable OBS may effectively protect against the development of ED, regardless of the underlying causes.

## Introduction

In andrology clinics, patients with erectile dysfunction (ED) are common. Patients usually complain about insufficient penile erection during sexual intercourse and seek advice about lifestyle and diet patterns^1^. According to predictions, ED might affect 322 million men by 2025 on a global scale^2^. It causes considerable psychological difficulties for male individuals, subsequently affecting their partner’s quality of life^3–5^. Although ED is not life-threatening, ED and physical health are intimately related; previous studies have proven that ED may be the precursor of CVD, dementia, Parkinson’s disease, and premature all-cause mortality^6–8^. To date, its incidence has risen in the young male population, although ED incidence is highly age dependent^9^. Therefore, proposing credible advice regarding preventing and alleviating ED is important.

Oxidative stress, which occurs when the oxidative balance is disrupted, reflects the excessive generation of reactive oxygen species (ROS) through the host’s protective antioxidant mechanism. Its relationship with erectile function has attracted increased interest from many researchers. Several epidemiological studies have identified systematic and regional oxidative stress in ED patients^10–12^, while other experimental studies with animal models have emphasized the therapeutic significance of oxidative stress^13–16^. From a clinical perspective, we hope for a transition from theory to clinical application. Therefore, we introduced a composite indicator calculated from nutrient intake and lifestyle factors: the oxidative balance score (OBS)^17^. Generally, a high OBS indicates a propensity for antioxidants rather than prooxidants, providing a comprehensive assessment of an individual's oxidative balance. If the independent correlation between OBS and ED can be validated in a large sample, it could offer potential value for guiding clinical practices. Therefore, we conducted this cross-sectional study to examine the association between dietary and lifestyle-integrated OBS and ED using high-quality data from the National Health and Nutrition Examination Survey (NHANES) 2001–2004. We hypothesized that OBS, which quantifies lifestyle and diet-derived oxidative stress, is negatively coupled with ED odds in the U.S. male adult population, independent of common risk factors for ED.

## Materials and method

### Data sources

The National Health and Nutrition Examination Survey (NHANES) is a continuous nationwide survey authorized by the National Center for Health Statistics (NCHS) Research Ethics Review Board. The NHANES provides a rich collection of variables and a considerable sample size, allowing for secondary analyses and generating nationwide representative conclusions. At the time of recruitment, all participants provided written consent. This study utilized only two survey cycles of data from NHANES (2001–2002 and 2003–2004) because a questionnaire asking about erectile status was available for only these two cycles. We strictly adhered tod the STROBE guidelines when reporting and interpreting our results^18^.

### Study population

**Figure 1:**
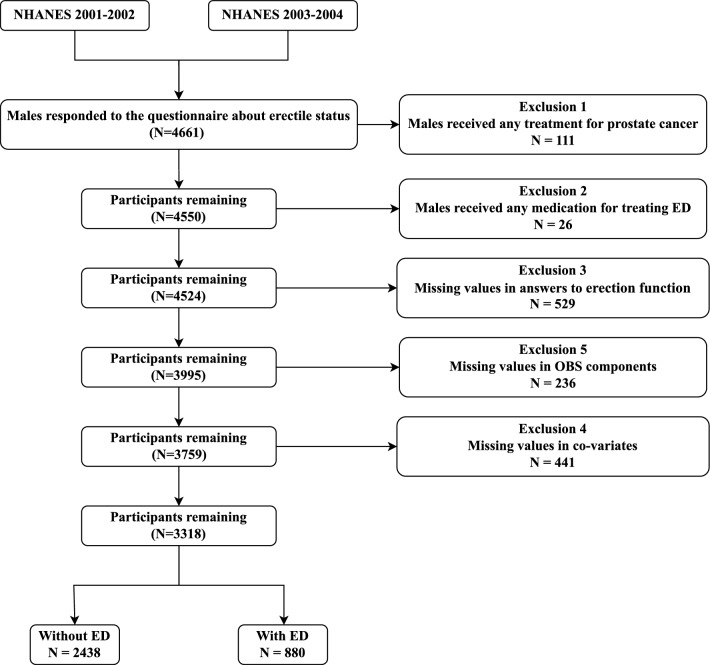
Flow diagram of the inclusion and exclusion of study participants.

The following specific criteria were utilized in the selection of study samples: 1. Must have provided an accurate response to the single-question erectile status evaluation; 2. Participants must be male and at least 20 years old; 3.Must not have undergone any treatment for prostate cancer; 4.Must not have been prescribed any phosphodiesterase type 5 inhibitory drugs (PDE5) or Yohimbine for erectile dysfunction; 5. Possess all the necessary data to calculate the OBS score. Ultimately, our final analysis included 3318 male participants, including 880 individuals with ED (Fig. 1). Approximately 11% of the original sample had missing values in covariables.

### Exposure

In the present study, we included 16 nutrients and 4 lifestyle factors, which included 5 prooxidants and 15 antioxidants, for the calculation of the OBS. To fully utilize the dietary recall data, based on the elements previously used in OBS^19^ calculations, we added 6 new components from dietary recall data: riboflavin^20^, niacin^21^, vitamin B6^22^, vitamin B12^23^, magnesium^24^, and copper^25^. A complete scheme outlining the assignment of scores can be found in Supplemental Table 1. The elements functioning as antioxidants include dietary fiber, carotene, riboflavin, niacin, vitamin B6, total folate, vitamin B12, vitamin C, vitamin E, calcium, magnesium, zinc, copper, selenium, total fat, iron, and physical activity. Each of these elements is assigned a score based on tertile values: the bottom tertile receives a score of 0, the middle tertile receives a score of 1, and the top tertile receives a score of 2. Conversely, the prooxidant elements consist of total fat, iron, alcohol, body mass index, and cotinine. The score assignments for total fat and iron intake are the inverse of those for the antioxidant elements, with the bottom tertile receiving a score of 2, the middle tertile receiving a score of 1, and the top tertile receiving a score of 0. In regard to alcohol consumption, those who are heavy drinkers (> 30 g/day) are assigned a score of 0, while mild-to-moderate drinkers (0–30 g/day) receive a score of 1, and non-drinkers are assigned a score of 2. When considering weight status, individuals classified as obese (BMI > 30) receive a score of 0, those classified as overweight (BMI 25–30) receive a score of 1, and those classified as normal weight (BMI ≤ 25) are allocated a score of 2.

### Outcome

In the NHANES, the evaluation of individuals’ erection function involved a single question, which inquired about their ability to achieve and sustain an erection that is sufficient for satisfactory intercourse. The response options provided were as follows: "able to do so always or almost always", "able to do so usually", "able to do so sometimes", or "unable to do so at all". This solitary inquiry was validated in a subgroup of participants from the Massachusetts Male Aging Study (MMAS), which demonstrated its utility as a pragmatic tool for identifying ED^28^. We defined those participants who indicated "able to do so sometimes" or "unable to do so at all" as our outcome of interest^26,27^.

### Covariables

Demographic features and potential confounders, which include age^28^, race^29^, poverty income ratio, education level^30^, marital status, hypertension^31^, diabetes^32^, cardiovascular diseases^31^, hypercholesterolemia^33^, and chronic kidney diseases^34,35^, were selected as covariables. In this study, participants were divided into three age groups: 20–40, 40–60, and older than 60 years. The race categories included Mexican American, non-Hispanic black, non-Hispanic white, other Hispanic, and other races. The poverty income ratio was classified into three categories: low income (≤ 1.3), medium income (> 1.3 to 3.5), and high income (> 3.5). Education was also categorized into three levels: high school or less, some college, and college graduate or higher (some college refers to individuals who have been awarded college credit but have not completed a degree). Marital status was analyzed based on three categories: Married/Living with Partner, Never married, and Widowed/Divorced/Separated. Hypertension was defined as taking antihypertensive agents, having a systolic blood pressure of ≥ 140 mmHg, or a diastolic blood pressure of ≥ 90 mmHg. Diabetes status was defined as a self-reported diabetes status, a fasting plasma glucose level of 126 mg/dl or greater, or a glycated hemoglobin level of 6.5% or greater. Cardiovascular disease (CVD) was determined based on participants' self-reported medical history of coronary heart disease, myocardial infarction, congestive heart failure, and stroke. Hypercholesterolemia was defined as having a total cholesterol level of ≥ 240 mg/dl or taking cholesterol-lowering drugs. The estimated glomerular filtration rate (eGFR) was calculated using the Chronic Kidney Disease Epidemiology Collaboration (CKD-EPI) equation, and chronic kidney disease (CKD) was identified as an eGFR less than 60.

### Statistical analysis

In this analysis, guidelines from the U.S. Centers for Disease Control and Prevention were followed to generate representative estimates^36^. To evaluate the discrepancies between the high- and low-OBS groups, we employed the chi-square test and U-test for categorical and continuous variables, respectively. Based on the intricate multistage probability sampling design of the NHANES data, we employed weighted logistic regression analysis from the "survey" package to estimate the adjusted impact of OBS on the odds of ED. When calculating the weight for this analysis, we referred to the official tutorial on weight calculation provided by the NHANES (https://wwwn.cdc.gov/nchs/nhanes/tutorials/Weighting.aspx). The sampling weight was calculated using the following formula: dietary recall 4-year combined weight = ifelse (survey year =  = '2001–2002', WTDRD/2, WTDR2D/2). We calculated the odds ratio (OR) along with its corresponding 95% confidence interval (CI) to assess the association. Furthermore, we evaluated both the continuous OBS variable and OBS tertiles to thoroughly examine its relationship with ED. We developed three models with varying degrees of adjustment. Model 1 was the crude model without any covariate adjustment, while Model 2 was a partially adjusted model that took into account common demographic factors, such as age, race, poverty income ratio, education, and marital status. Finally, Model 3 was the fully adjusted model that incorporated all covariates. To assess the trend, we calculated P for trend by introducing the median value of OBS in each OBS tertile as a continuous variable in the respective models. Furthermore, we used generalized additive model regression (GAM) to explore the potential nonlinear association between OBS and ED. The generalized additive model (GAM) is a proper tool for investigating real-world nonlinear correlations. This allows us to fit a model with a nonlinear smoothing term. Because the connections between variables in additive models are nonparametric, they are typically not characterized by simple mathematical formulas (such as y = ax + b in linear regression). As a result, graphically smoothed curves are often employed in GAM to observe variable dependencies and thus to detect possible response linkages based on the curve's shape. In this analysis, we used the R package "mgcv" to build a GAM for predicting ED using the smoothing term of OBS and adjusted for all covariates. The degree of smoothness of the fitted curve can be altered using the smoothing term's degrees of freedom (df). We select the best df using the minimal generalized cross validation (GCV) approach. We also conducted a subgroup analysis for the selected variables. We calculated the P value for interactions by comparing the models with or without the interaction term (continuous OBS * effect modifier) using the likelihood ratio test.

Three sensitivity analyses were also conducted to ensure the robustness of our results. In the first sensitivity analysis, we further adjusted the HEI-2015 score in the full model because dietary quality could affect the dietary intake of antioxidants and prooxidants^37^. In the second analysis, only those who responded “unable to do so at all” were considered to have ED. To account for missing values for some covariables, a third sensitivity analysis was performed. Using the R package "Jomo"^38^, missing values for covariables were imputed based on the multiple imputation method. Five imputed datasets were created and used for weighted logistic regression analysis.

In all analyses, a two-tailed P < 0.05 was considered to indicate statistical significance. All the statistical analyses were performed with the statistical software R 4.3.1.

## Results

### Baseline characteristics

A total of 3318 participants, including 880 individuals with erectile dysfunction (ED), were included in the analysis. Table 1 presents the characteristics of the participants. OBS scores ranged from 3 to 36 points, and the study sample was assigned to high- or low-OBS groups based on the median value of OBS. Compared with those in the low OBS group, those in the high OBS group had a greater incidence of ED. (30.99% vs 21.6%, P value < 0.001). In addition, participants with higher OBS were more likely to be 20–40 years old (38.89% vs 29.67%, P value < 0.001), Non-Hispanic White(60.29% vs 50.03%, P value < 0.001), have a poverty income ratio higher than 3.5 (43.45 vs 32.32, P value < 0.001), have an educational level of college graduate or higher(27.42 vs 16.33, P value < 0.001), have marriage or live-partner(71.75 vs 68.60, P value = 0.013), have lower prevalence of hypertension(33.76 vs 44.80, P value = 0.013), diabetes(11.46 vs 15.99, P value ≤ 0.001), cardiovascular disease (10.96 vs 14.49, P value = 0.003), hypercholesterolemia(26.35 vs 30.59, P value = 0.008), and chronic kidney disease(6.14 vs 10.64, P value ≤ 0.001).

**Table 1:** Baseline characteristics of participants from 2001 to 2004 National Health and Nutrition Examination Survey (NHANES), divided by oxidative balance score tertiles.

Variables	Oxidative balance score by tertiles			P value
	Overall	Low [3, 20]	High (20, 36]	
Number of participants	3318	1739	1579	
OBS (mean (SD))	19.97 (7.24)	14.10 (3.95)	26.42 (3.66)	< 0.001
ED (%)				< 0.001
No	2438 (73.48)	1200 (69.01)	1238 (78.40)	
Yes	880 (26.52)	539 (30.99)	341 (21.60)	
Age (%)				< 0.001
[20, 40)	1130 (34.06)	516 (29.67)	614 (38.89)	
[40, 60)	1128 (34.00)	598 (34.39)	530 (33.57)	
[60, )	1060 (31.95)	625 (35.94)	435 (27.55)	
Race (%)				< 0.001
Mexican American	690 (20.80)	358 (20.59)	332 (21.03)	
Non-Hispanic Black	584 (17.60)	390 (22.43)	194 (12.29)	
Non-Hispanic White	1822 (54.91)	870 (50.03)	952 (60.29)	
Other Hispanic	119 ( 3.59)	66 ( 3.80)	53 ( 3.36)	
Others	103 ( 3.10)	55 ( 3.16)	48 ( 3.04)	
Poverty income ratio (%)				< 0.001
≤ 1.3	777 (23.42)	478 (27.49)	299 (18.94)	
1.4–3.5	1293 (38.97)	699 (40.20)	594 (37.62)	
> 3.5	1248 (37.61)	562 (32.32)	686 (43.45)	
Education (%)				< 0.001
High school or less	1720 (51.84)	996 (57.27)	724 (45.85)	
Some college	881 (26.55)	459 (26.39)	422 (26.73)	
College graduate or higher	717 (21.61)	284 (16.33)	433 (27.42)	
Marital status (%)				0.013
Married/living with partner	2326 (70.10)	1193 (68.60)	1133 (71.75)	
Never married	554 (16.70)	288 (16.56)	266 (16.85)	
Widowed/divorced/separated	438 (13.20)	258 (14.84)	180 (11.40)	
Hypertension (%)				< 0.001
No	2006 (60.46)	960 (55.20)	1046 (66.24)	
Yes	1312 (39.54)	779 (44.80)	533 (33.76)	
Diabetes (%)				< 0.001
No	2859 (86.17)	1461 (84.01)	1398 (88.54)	
Yes	459 (13.83)	278 (15.99)	181 (11.46)	
CVD (%)				0.003
No	2893 (87.19)	1487 (85.51)	1406 (89.04)	
Yes	425 (12.81)	252 (14.49)	173 (10.96)	
Hypercholesterolemia (%)				0.008
No	2370 (71.43)	1207 (69.41)	1163 (73.65)	
Yes	948 (28.57)	532 (30.59)	416 (26.35)	
CKD (%)				< 0.001
Yes	282 ( 8.50)	185 (10.64)	97 ( 6.14)	
No	3036 (91.50)	1554 (89.36)	1482 (93.86)	

Data are presented as percentages for categorical variables and means ± SD for continuous variables.

*ED* erectile dysfunction, *CVD* cardiovascular diseases, *OBS* oxidative balance score, *CKD* chronic kidney diseases.

### Weighted logistic regression and GAM analysis

**Figure 2:**
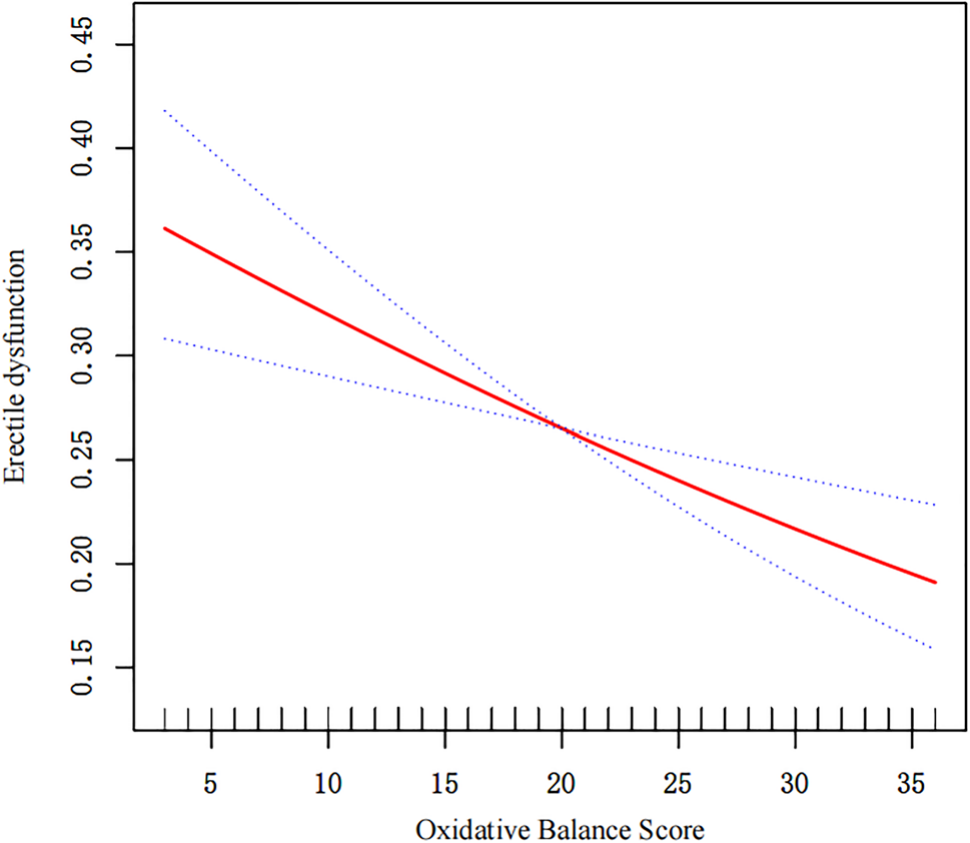
Graphics of smooth curve fittings between the OBS and ED. Blue bands represent the 95% CI from the fit. The solid red line represents the smooth curve fit between variables. This model was adjusted for age, race, poverty income ratio, education and marital status, hypertension, diabetes, cardiovascular disease, hypercholesterolemia and chronic kidney disease.

Table 2 shows the findings of three logistic regression models that consistently reveal a stable estimate of the correlation between OBS and ED, even after adjusting for various factors. According to the initial model (Model 1), which did not include any covariates, the odds ratio (OR) of continuous OBS measurements at the ED was 0.96 (95% CI 0.94–0.97). Notably, individuals in higher OBS tertiles had relatively lower odds of having ED than did those in lower OBS tertiles (OR = 0.47, 95% CI 0.33–0.67). Model 2 accounted for demographic factors such as age, race, the poverty income ratio, education, and marital status, yet the odds ratio remained at 0.96 (95% CI 0.94–0.98). For OBS tertiles 2 and 3, the odds ratios were 0.65 (95% CI 0.49–0.87) and 0.56 (95% CI 0.38–0.82), respectively, indicating a significant decreasing trend (p for trend = 0.005). The fully adjusted model (Model 3) further considered hypertension, diabetes, CVD incidence, hypercholesterolemia, and CKD, for which the odds ratio was 0.96 (95% CI 0.94–0.98). Notably, the odds ratios for OBS tertiles 2 and 3 decreased to 0.67 (95% CI 0.50–0.90) and 0.57 (95% CI 0.37–0.87), respectively, and the trend remained robust (p for trend = 0.012). Furthermore, Fig. 2 demonstrates the compelling linear association between OBS and ED through GAM analysis, which adds more weight to the evidence presented in Table 2.

**Table 2:** The relationship between OBS and ED descripted by weighted logistic regression.

Exposure	Model 1		Model 2		Model 3	
	OR	95% CI	OR	95% CI	OR	95% CI
OBS (continious)	0.96	0.94, 0.97	0.96	0.94, 0.98	0.96	0.94, 0.98
OBS (tertiles)						
Q1 [3, 16]	Reference		Reference		Reference	
Q2 (16,24]	0.72	0.56, 0.93	0.65	0.49, 0.87	0.67	0.50, 0.90
Q3 (24,36]	0.47	0.33, 0.67	0.56	0.38, 0.82	0.57	0.37, 0.87
P for trend	< 0.001		0.005		0.012	

Model 1: No covariates were adjusted; Model 2: Age, race, poverty income ratio, education and marital status were adjusted; Model 3: Age, race, poverty income ratio, education and marital status, hypertension, diabetes, cardiovascular diseases, hypercholesterolemia, and chronic kidney diseases were adjusted. *OBS* oxidative balance score, *OR* odd ratio, *CI* confidential interval.

### Subgroup and interaction analysis

**Figure 3:**
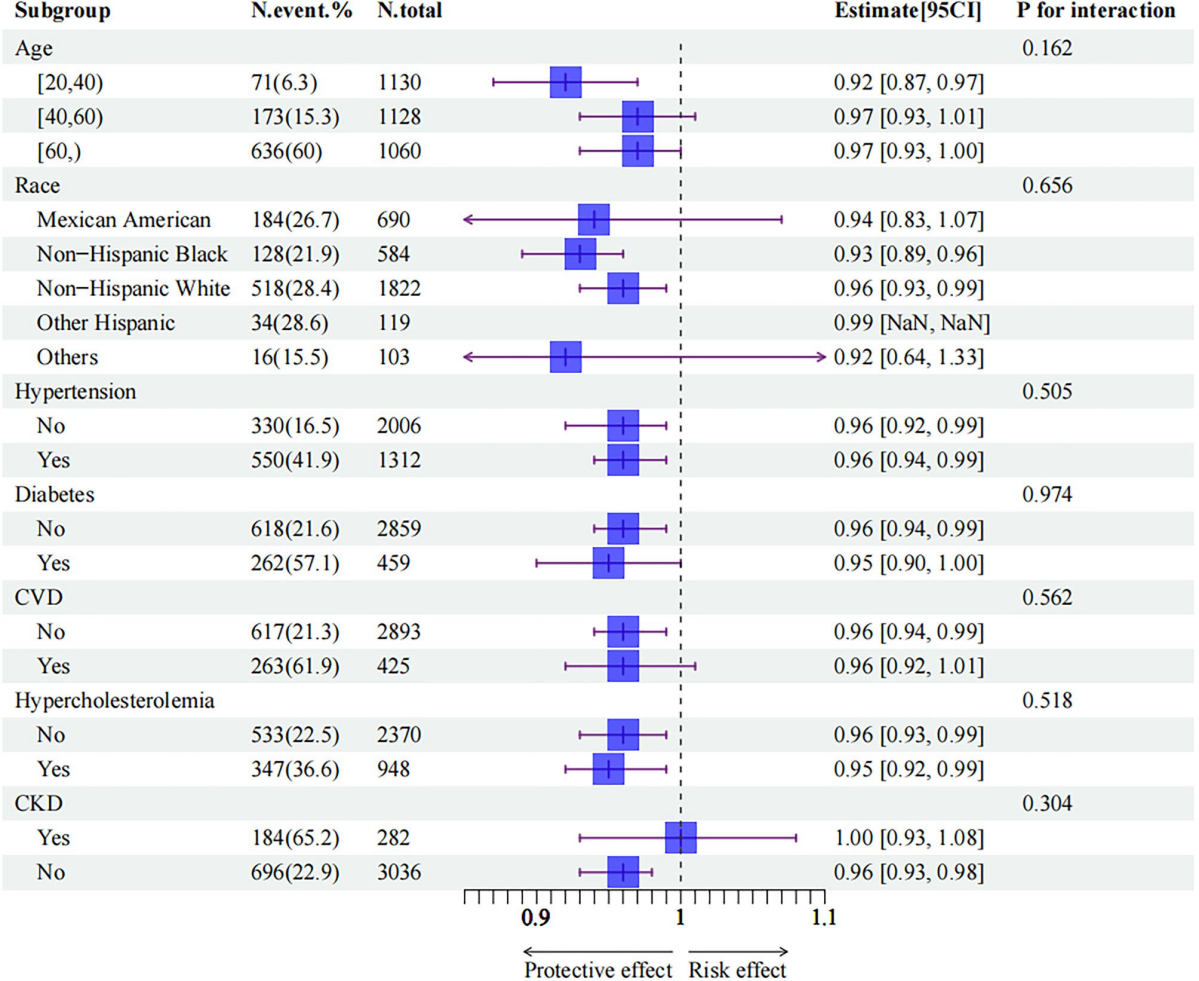
Subgroup and interaction analysis of the relationship between OBS and ED. Each stratification was adjusted for age, race, poverty income ratio, education and marital status, hypertension, diabetes, cardiovascular disease, hypercholesterolemia and chronic kidney disease, except the stratification factor itself. In the subgroup of race, the group of other Hispanic have no estimate because the model cannot converge.

To investigate the consistency of the relationship between OBS and ED in the general population and explore potential variations among different population subsets, subgroup analysis and interaction tests were conducted. The findings among the distinct subgroups based on specific variables are visually presented in Fig. 3. Among participants aged > 20 to 40 years, there was a significant association between continuous OBS and ED, with an odds ratio of 0.92 (95% CI 0.87–0.97). Similar estimates were observed across various subgroups stratified by race, hypertension, diabetes status, CVD incidence, hypercholesterolemia, and CKD incidence. It is worth noting that while some of these estimates did not reach statistical significance, this could be attributed to a reduction in statistical power. Moreover, the interaction analysis did not reveal any significant effects, suggesting that the relationship between OBS and ED does not differ significantly among the examined subgroups.

### Sensitivity analysis

Table 3 presents the results of three sensitivity analyses conducted to further explore the relationship between OBS and ED. In the first sensitivity analysis, we included the HEI-2015 score in the fully adjusted model to account for diet quality. The odds ratio for continuous OBS remained consistent at 0.96 (95% CI 0.93–0.99), indicating a significant association. Moreover, participants in higher OBS tertiles had a decreased odds of experiencing ED compared to those in lower OBS tertiles, with an odds ratio of 0.56 (95% CI 0.34–0.91). For the second sensitivity analysis, only participants who responded "never able" were classified as having ED, resulting in a total of 326 participants in this category. The odds ratio for continuous OBS on ED was calculated to be 0.96 (95% CI 0.93–1.00), suggesting a similar association as that observed in the previous analyses. Participants in higher OBS tertiles exhibited a greater odd of having ED than those in lower OBS tertiles, with an odds ratio of 0.48 (95% CI 0.24–0.94). For the third sensitivity analysis, pooled regression coefficients from 5 datasets generated by multiple imputation also yielded similar conclusion.

**Table 3:** Sensitivity analysis.

Exposure	Sensitivity analysis 1		Sensitivity analysis 2		Sensitivity analysis 3	
	OR	95% CI	OR	95% CI	OR	95% CI
OBS (continious)	0.96	0.93, 0.99	0.96	0.93, 1.00	0.96	0.94,0.98
OBS (tertiles)						
Q1 [3, 16]	Reference		Reference		Reference	
Q2 (16,24]	0.66	0.47, 0.91	0.8	0.47, 1.34	0.72	0.52, 0.98
Q3 (24,36]	0.56	0.34, 0.91	0.48	0.24, 0.94	0.60	0.41, 0.86
P for trend	0.039		0.017		< 0.05*	

Sensitivity analysis 1: The model was adjusted for age, race, poverty income ratio, education and marital status, hypertension, diabetes, cardiovascular disease, hypercholesterolemia, chronic kidney disease and HEI-2015 score; Sensitivity analysis 2: Having ED was redefined to those who responded “never able”. The model was adjusted for age, race, poverty income ratio, education and marital status, hypertension, diabetes, cardiovascular disease, hypercholesterolemia, chronic kidney disease and HEI-2015 score. Sensitivity analysis 3: Missing data in covariables were multiply imputed using a multilevel approach designed for survey data. 5 imputed datasets were generated and used for regression analysis. The pooled regression coefficients were calculated based on Rubin rules. The model was adjusted for age, race, poverty income ratio, education and marital status, hypertension, diabetes, cardiovascular disease, hypercholesterolemia, chronic kidney disease.

*Specific pooled P-value is not available.

## Discussion

To our knowledge, this is the study with the largest sample size that demonstrates an independent relationship between OBS and ED. According to the crude model, participants in the highest OBS tertiles still had approximately 50% greater odds of having ED than those in the lowest OBS tertiles. After taking into account some common demographic factors and known ED risk factors, the regression coefficient did not obviously change among the models, and the linear trend also remained stable. Upon employing the GAM (Fig. 2) to model the relationship between OBS and ED, we identified a stable linear relationship, with no evidence of the expected non-linearity. In the subgroup analysis, although some 95% CIs included one due to the insufficient sample size, the estimates of most subgroups remained relatively unchanged. As shown in Fig. 3, in the age subgroup, younger males may have a more significant protective effect of OBS on ED, although the interaction P values were not significant. After considering the impact of diet quality and restricting the outcome to severe ED, our results remained solid. To conclude, the present study provides a preliminary evidence of a robust negative relationship between OBS and ED.

Oxidative stress plays a pivotal role in the development of ED. In response to sympathetic nerve cholinergic stimulation, nitric oxide (NO) is produced by corpus cavernosum endothelial cells, leading to increased synthesis of cyclic GMP (cGMP), which in turn relaxes smooth muscle and facilitates erection^39^. Therefore, any disruption of this mechanism may contribute to ED. Under normal circumstances, various antioxidant enzymes and antioxidants maintain an optimal concentration of reactive oxygen species (ROS), thus preserving oxidative balance. However, when ROS production exceeds normal levels or when antioxidant availability decreases, oxidative stress occurs^40^. Excessive ROS impair NO production^41^ and lead to both neurogenic and vasculogenic dysfunction, ultimately resulting in ED^42^. Additionally, the interaction between NO and excessive ROS can generate peroxynitrite, which inactivates superoxide dismutase (SOD) and establishes an intensified positive feedback loop^43^. Despite the promising effects of phosphodiesterase-5 (PDE5) inhibitors in symptomatically treating ED through the inhibition of cGMP hydrolysis to GMP, these drugs are costly and may induce unexpected side effects. Moreover, these methods fail to address the progression of cardiovascular and neurological complications, which are common manifestations of systemic oxidative stress^7,44–46^. Therefore, restoring systemic oxidative balance may be a preferable approach for the treatment and prevention of ED.

Several studies have investigated the impact of oxidative stress on erectile dysfunction (ED). However, the results from two cross-sectional studies examining the relationship between serum markers of oxidative stress and ED are conflicting. In a 2017 cross-sectional study by Thierry et al. a negative correlation was found between IL-18 and IL-8 levels and International Index of Erectile Function (IIEF) score, independent of factors such as age, BMI, smoking status, diabetes status, adiponectin, Mox-LDL, and testosterone^11^. Conversely, a 2022 cross-sectional study by Fujita et al. did not find a significant correlation between serum markers of oxidative stress and IIEF scores after adjusting for variables such as age, hypertension, diabetes, calcium blocker usage, β-blocker usage, the Brinkman index, and the eGFR^10^. These inconsistent findings may be attributed to variations in sample sizes, study populations, or adjusted covariates. Similarly, the CKD subgroup in our study also yielded nonsignificant results. Another cross-sectional study by Barassi et al. indicated that disruption of the oxidative balance may play a more significant role in arteriogenic ED than in nonarteriogenic ED^12^. In addition, several experimental studies have confirmed that certain drugs and stem cell therapies have encouraging therapeutic effects on animal models by inhibiting oxidative stress. Unfortunately, the conclusions drawn from these epidemiological and experimental studies may not be universally applicable, and some findings may possess limited clinical translational significance.

Our study established a strong association between OBS and ED incidence, independent of the considered risk factors. A higher OBS, indicating greater antioxidant potential, was associated with a reduced risk of ED. Furthermore, some acknowledged risk factors for ED do not seem to be mediators or confounding factors in this relationship. The results from the GAM did not indicate a threshold effect or non-linear relationship between the OBS and ED, as excessive intake of certain antioxidants can be harmful or even toxic. Our findings suggest that OBS may offer a robust computational approach, whereby achieving certain dietary or behavioral standards that elevate the OBS score could yield substantial health benefits. These findings align with previous research focusing on specific components of OBS. Specifically, Muniz highlighted how alcohol impairs erectile function by elevating intracellular ROS and reducing SOD activity in the corpus cavernosum^16^. Helmy demonstrated that supplementation with vitamin E improves NO levels in rat penile tissue^47^. Furthermore, Tostes emphasized the role of oxidative stress in the negative impact of smoking on erectile function^48^. In a cross-sectional study involving 134 ED patients and 50 healthy controls, Jiangnan reported a greater prevalence of low blood levels of folic acid and vitamin B12 among ED patients than among control individuals^49^. Similarly, Liu's study of 3745 adult males in the U.S. revealed a link between ED risk and the intake of antioxidants such as magnesium, zinc, copper, and selenium^50^. However, these studies did not fully consider the influence of prooxidants or the synergistic effects of antioxidants within biological systems^51^. Notably, a recent meta-analysis by Su et al. highlighted the combined benefits of PDE5 inhibitors and antioxidant supplements^52^. Overall, adopting a dietary and lifestyle pattern that supports OBS could offer protective and therapeutic effects against ED.

While previous studies have provided evidence of increased oxidative stress markers in patients with ED^10–12^, the exact mechanism by which OBS affects ED is not fully understood. One plausible explanation is that a dietary and lifestyle pattern promoting OBS may elevate serum antioxidant levels, leading to a collaborative biological effect. This collaborative effect could reduce the production of reactive oxygen species (ROS), mitigate the systemic inflammatory response^53^, and consequently alleviate damage to endothelial cells in the corpus cavernosum^54,55^. From a clinical perspective, ED can be classified into psychogenic and organic causes. Psychogenic ED is primarily attributed to factors such as depression and anxiety, while organic ED is mainly associated with vascular, hormonal, and neurogenic factors^9^. Encouragingly, OBS has been found to be inversely related to depression^56^, CKD^57^, diabetes^58,59^, and hypertension^60^. According to our subgroup analysis, the protective effect of OBS, although not significantly, was more prominent in the 20–40 age group than in the other age groups. These findings suggest that OBS may still offer protective effects against ED associated with psychogenic factors, which are the predominant causes of ED in this age group^9^. Based on the concept of oxidative balance in the etiology of ED and the existing evidence, we propose that adopting a lifestyle and dietary pattern that supports OBS may have a protective effect against the development of ED, regardless of the specific clinical cause. Therefore, when faced with inquiries regarding improving ED through health behavior and diet, health consultants or clinical physicians can confidently recommend the adoption of an OBS-facilitating approach based on our robust conclusion.

There are several strengths in our study that contribute to the reliability and applicability of our findings. First, we utilized high-quality data from the NHANES, which incorporates survey design into regression analysis, enhancing the generalizability of our conclusions to the male population in the U.S. and similar developed, multiracial countries. Second, we comprehensively considered a range of dietary and lifestyle variables when calculating OBSs, allowing for the translation of our findings into meaningful health advice. However, there are also limitations in our study that need to be acknowledged. First, instead of using the International Index of Erectile Function (IIEF) scale, we relied on the Massachusetts Male Aging Study (MMAS) single question to identify ED. Although the MMAS single question has demonstrated high predictive accuracy in predicting ED through urologic examination, with an area under the curve of 0.888, it may still have inherent limitations compared to a comprehensive assessment using the IIEF scale^61^. Second, the data used for analysis in our study were not up-to-date. Our results still require further validation with more recent data. Third, some essential variables were obtained through recall interviews, introducing potential recall bias into our analysis. Fourth, due to the cross-sectional study design, we cannot establish causal relationships between OBS and ED. Therefore, further prospective studies are needed to explore the temporal relationship between OBS and ED development. Notably, excessive intake of certain antioxidants may have adverse effects or toxicity, which was not considered in our OBS calculations. Careful attention should be given to the safety thresholds of antioxidant intake to avoid potential harm. Finally, despite adjusting for a wide range of ED risk factors in our comprehensive model, there may still be unmeasured or residual confounding factors, including psychological disorders, that could influence the association between OBS and ED. However, the consistent association between OBS and ED, as demonstrated by stable estimates in various adjusted models and logical reasoning, suggests that OBS is less likely to be significantly impacted by unmeasured factors.

## Conclusion

In the present study, we found a robust relationship between OBS and ED in nationally representative data, which was more significant for younger men. This adverse connection implies that, regardless of the specific cause, a lifestyle and diet pattern that promotes high OBS may be useful for preventing the development of ED. However, further research in this area is necessary to establish causality.

### Supplementary Information

Supplementary Table 1.

## Data Availability

The original contributions presented in the study are included in the article/Supplementary Material, further inquiries can be directed to the corresponding author/s.

## Abbreviations

ED: Erectile dysfunction
OBS: Oxidative balance score
NHANES: National Health and Nutrition Examination Survey
CVD: Cardiovascular disease
ROS: Reactive oxygen species
CKD: Chronic kidney disease
OR: Odds ratio
CI: Confidence interval
NO: Nitric oxide
