# Supplementary material for: The relationship between oxidative balance score and erectile dysfunction in the U.S. male adult population
Source: Sci Rep. 2024 May 10;14:10746. doi: 10.1038/s41598-024-61287-w (PMC11087471; doi:10.1038/s41598-024-61287-w)
Supplement: Supplementary file 1 — Supplementary Table 1. [file 41598_2024_61287_MOESM1_ESM.pdf]

Supplemental table 1 Oxidative balance score assignment scheme.

OBS Components	Property	Assignment Scheme *
**Dietary components**		
1. Dietary fiber (g/d)	Antioxidant	0 = low (1st tertile), 1 = intermediate (2nd tertile), 2 = high (3rd tertile)
2. Carotene (RE/d)		
3. Riboflavin (mg/d)		
4. Niacin (mg/d)		
5. Vitamin B6 (mg/d)		
6. Total folate (mcg/d)		
7. Vitamin B12 (mcg/d)		
8. Vitamin C (mg/d)		
9. Vitamin E (ATE) (mg/d)		
10. Calcium (mg/d)		
11. Magnesium (mg/d)		
12. Zinc (mg/d)		
13. Copper (mg/d)		
14. Selenium (mcg/d)		
15. Total fat (g/d)	Prooxidant	0 = high (3rd tertile), 1 = intermediate (2nd tertile), 2 = low (1st tertile)
16. Iron (mg/d)		
**Lifestyle components**		
17. Physical activity (MET-minute/week)	Antioxidant	0 = low (< 400), 1 = intermediate (400 –1000), 2 = high (>1000)
18. Alcohol (g/d)	Prooxidant	0 = high (> 30), 1 = intermediate (0 – 30), 2 = low (None)
19. BMI (kg/m2)		0 = high (> 30), 1 = intermediate (25 – 30), 2 = low (< 25)
20. Cotinine (ng/mL)		0 = high (> 0.038), 1 = intermediate (0.038 – 1.13), 2 = low (< 1.13)

BMI: body measurement index.
